# Production of volatile fatty acids through microbial conversion of waste sludges: influence of substrates

**DOI:** 10.1186/s40643-026-01049-w

**Published:** 2026-05-08

**Authors:** Lei Liu, Jenni Salminen, Taina Lundell, Martin Romantschuk, Merja Hannele Kontro

**Affiliations:** 1 Faculty of Biological and Environmental Sciences, University of Helsinki, Niemenkatu 73, 15140 Lahti, Finland; 2 Department of Microbiology, Faculty of Agriculture and Forestry, University of Helsinki, Viikki Biocenter 1, Viikinkaari 9, 00014 Helsinki, Finland

**Keywords:** Volatile fatty acids, Waste sludge valorisation, Microbial community, Sludge fermentation, Bioprocess sustainability, Resource recovery

## Abstract

Inadequate sludge treatment poses a significant risk of environmental pollution. To reduce pollution, utilizing waste sludge as an energy source offers a sustainable solution to mitigate pollution. Since sludges contain abundant organics, they are expected to produce more valuable organics, such as volatile fatty acids (VFAs). Although sludge fermentation has been widely studied, direct yields of VFA production from different sludge types are still limited, particularly for sludge materials originating from Finnish wastewater treatment plants. In this study, laboratory-scale bioreactors were built to examine VFA production from digester feed sludge, digested sludge, and ammonia removal sludge, which were collected from local wastewater treatment plants. Among the tested substrates, digested feed sludge resulted in the highest VFA yield, reaching 171.6 ± 6.3 mg/g volatile solids after 11 days of incubation, which indicated its superior potential for VFA generation. In contrast, bioreactors fed with digested sludge or ammonia removal sludge showed no clear increasing trend in VFA production. The addition of sawdust led to lower overall VFA yields, approximately half of those obtained with digested feed sludge alone, and increasing the sludge proportion did not result in further yield enhancement. Acetic acid was the dominant VFA in all bioreactors, accounting for 61.2–97.9% of total VFAs. Microbial community analysis indicated the prevalence of phyla Bacillota and Pseudomonadota, with *Lactobacillus* being relatively abundant in bioreactors exhibiting higher VFA production. These findings suggest that sludge type plays an important role in determining VFA production performance and may support more informed selection of sludge substrates for VFA-oriented sludge valorisation.

**Graphical Abstract:**

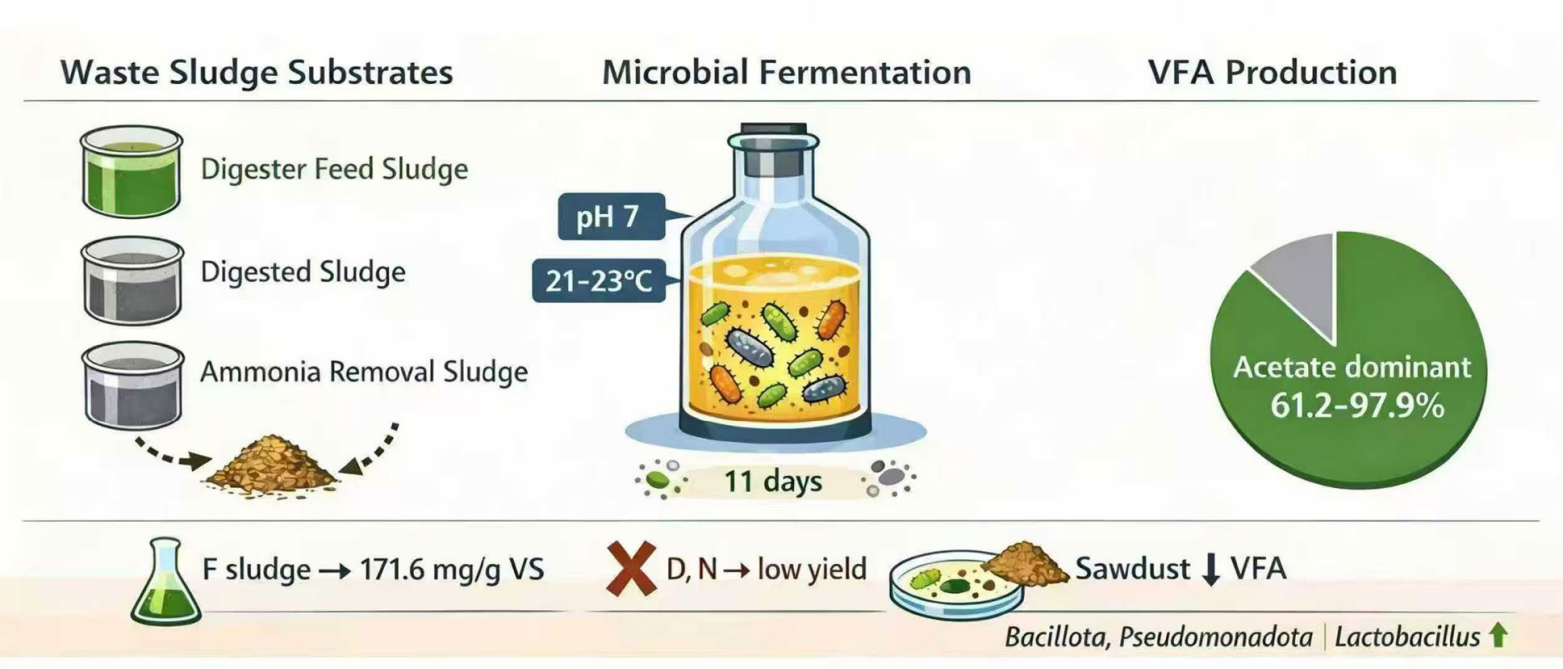

## Highlights

Digester feed sludge exhibited the highest VFA production potential.

Sawdust addition did not enhance VFA yields in sludge-based bioreactors.

Digested sludge and ammonia removal sludge showed limited VFA production.

Acetic acid dominated the VFA profile in all bioreactors.

Bacillota and Pseudomonadota dominated the microbial communities.

## Introduction

Volatile fatty acids (VFAs) are short-chain fatty acids containing two to six carbon atoms, including acetic acid, propionic acid, butyric acid, valeric acid, and caproic acid (Bergman 1990; Lee et al. 2014). Owing to their chemical versatility, VFAs are key industrial feedstocks and raw materials in pharmaceutical and chemical industries (Varghese et al. 2022). In particular, VFAs serve as renewable carbon sources for the synthesis of polyhydroxyalkanoates (PHAs), a class of biodegradable polymers that offer a sustainable alternative to petroleum-derived plastics and reduce environmental pollution (Morgan-Sagastume et al. 2015; Sharma et al. 2021). VFAs also play an essential role as substrates in biological nitrogen and phosphorus removal from wastewater and anaerobic digestion for biogas production (Liu et al. 2018; James et al. 2021).

Despite their broad applicability, most VFAs are currently produced chemically (Bhatia and Yang 2017). Biological VFA production has attracted increasing attention as a sustainable alternative, particularly in the context of rising energy costs (Lee et al. 2014). However, industrial VFA production commonly relies on sugars as carbon sources, which raises both economic and ethical concerns, the latter due to competition with food sources (Bhatia and Yang 2017). Consequently, the use of waste-derived substrates for VFA production has emerged as a key research focus within the field of wastewater resource recovery. In addition, Recent studies have also explored various strategies for wastewater treatment and resource recovery, including microbial lipid production and integrated anaerobic treatment processes (Al Shehhi et al. 2025; Mazaheri et al. 2024).

Activated sludge has been widely used during wastewater treatment process since the early 20th century (Fang et al. 2020). This method has become a widely used biological technology for the purification of wastewater and the removal of nutrients and harmful microbes. A major drawback of this process is the generation of large quantities of excess sludge, which poses significant economic and environmental challenges. Sludge contains substantial amounts of organic matter and potentially recoverable resources. However, its reuse is often constrained by the presence of contaminants such as chemicals, heavy metals, and other hazardous compounds (Fytili and Zabaniotou 2008; Kouzi et al. 2020). Additionally, wastewater treatment plants (WWTPs) allocate more than half of their operational budget to treating the sludge (Presti et al. 2021), highlighting the need for alternative sludge management strategies that enable value recovery.

VFAs are produced from waste sludge based on a mixed hydrolysis process involving both chemical and biological reactions. The production yield and distribution of VFAs are influenced by operational conditions, such as pH, temperature, organic substrate materials, and carbon-to-nitrogen ratio (C/N) (Jiang et al. 2013). Among these factors, pH is particularly important for the bioreactors (Lin and Li 2018). Although enhanced VFA yields have been reported under alkaline conditions (pH 8–11) (Fang et al. 2020), the operation at neutral pH has been shown to support the more effective acidogenesis and the overall VFA production while reducing chemical demand (Lukitawesa et al. 2020; Ma et al. 2016).

Temperature also plays a key role in influencing the production and composition of VFAs. In general, hydrolysis bioreactors for VFA production from wastewater sludge have been operated under psychrophilic (< 20 °C) and mesophilic (25–40 °C) to thermophilic (45–60 °C) conditions (Al-Sulaimi et al. 2022). A controlled increase in bioreactor operation temperature up to 45 °C has enhanced the production of VFAs (Jiang et al. 2013). However, the activities of acidification enzymes are higher under mesophilic conditions (Nie et al. 2021). Moreover, higher temperatures increase the pKa of VFAs, promoting the undissociated form, which can inhibit microbial activity through membrane diffusion and intracellular acidification (Sukphun et al. 2021). From an energy-efficiency and process-stability perspective, operation at room temperatures (approximately 21 to 23 °C) in this study is therefore attractive for practical implementation.

Typically, the C/N of sludge ranges from 6 to 9, which may limit acidogenic performance (Fang et al. 2020). Supplementation with carbon-rich co-substrates has been shown to enhance VFA production by improving the C/N balance (Ma et al. 2017). Considering the economic and environmental factors, sawdust represents a low-cost and widely available carbon source, particularly in Nordic regions, where sawdust is generated in large quantities as a by-product of the wood-processing industry.

In the present study, waste sludge bioreactors were operated aerobically by using digester feed sludge, digested sludge, and ammonia removal sludge obtained from local municipal WWTPs and company as carbon substrates for VFA production in Lahti, Finland. The objectives were to (i) evaluate and compare the VFA production potential of different sludge types under aerobic conditions and (ii) assess differences in microbial community composition associated with VFA generation.

## Materials and methods

### Waste materials

Waste materials used in this study were acquired from local wastewater treatment plants and companies in Finland. Ammonia removal sludge by stripping was obtained from Gasum Ltd (Espoo, Finland); activated sludge and digested sludge were from Lahti Aqua Ltd (Lahti, Finland); digester feed sludge and compost were obtained from Labio Ltd (Lahti, Finland). Digester feed sludge was a mixture of source-separated household biowaste and wastewater sludge in a volume ratio of 2:1 (vol/vol). The 6-day-old compost was a mixture of garden waste, source-separated household biowaste, and digested sludge. Sawdust was obtained from Salpakierto Ltd (Lahti, Finland). Material properties are summarized in Table 1.

**Table 1:** Main properties of waste materials in this study

Parameter	Digester feed sludge	Digested sludge	Ammonia removal sludge	Activated sludge	Compost	Sawdust
Volatile solids (mg/g)	938 ± 2	590 ± 3	886 ± 4	634 ± 19	562 ± 27	996 ± 1.2
Fixed solids (mg/g)	62 ± 2	410 ± 3	114 ± 4	366 ± 19	438 ± 27	3.8 ± 1.2
Total-C (mg/g)	487 ± 29	278 ± 1	487 ± 16	303 ± 2.6	311 ± 19	473 ± 3
Total-N (mg/g)	27 ± 3	47 ± 1	81 ± 3	65 ± 1	35 ± 4	0.08 ± 0.1
C/N	18.1 ± 0.4	6.0 ± 0.1	6 ± 0.3	4.7 ± 0.01	8.8 ± 0.4	5835 ± 0.3

### Experimental setup and operation

In this study, aerobic laboratory-scale hydrolysis bioreactors were set up in 1000 mL glass laboratory bottles separately in triplicate using either digester feed sludge, digested sludge, or ammonia removal sludge as substrate and sawdust as an additive (Table 2). Each bioreactor contained an inoculum, which consisted of activated sludge, digester feed sludge, digested sludge, and compost, all calculated based on 0.3 g of dry weight (DW) basis to provide a functionally diverse microbial community. Volatile solids (VS) of the sludge were adjusted to 25 g in bioreactors without and with sawdust (Table 2). Additionally, in bioreactors (BLFS) and (BLNS) using digester feed sludge and ammonia removal sludge, the sludge content was reduced to 9.1 g and sawdust (15.9 g) was added to keep VS at 25 g (Table 2). Bioreactors in 1 L flasks were shaken at 180 rpm (Edmund Bühler GmbH, Bodelshausen, Germany). pH was maintained at 7 by daily adjustment using 4.0 M sodium hydroxide. A 3.0 mL supernatant sample was collected daily from each bioreactor and stored at -20 °C in a freezer for subsequent analyses.

**Table 2:** Bioreactor supplements and compositions in this study

	Digester feed sludge			Digested sludge		Ammonia removal sludge		
	BF	BFS	BLFS	BD	BDS	BN	BNS	BLNS
Bioreactor supplements								
Sludge (g DW)	26.7	26.7	9.2	42.4	42.4	28.2	28.2	10.1
Sawdust (g DW)	0	16	16	0	16	0	16	16
Bioreactor compositions								
VS (g)	25.0	25.0 + 15.9	9.1 + 15.9	25.0	25.0 + 15.9	25.0	25.0 + 15.9	9.1 + 15.9
VS (% of DW)	92.7	95.2	96.2	59.2	70.1	87.8	92.0	94.2
FS (% of DW)	7.3	4.8	3.8	40.8	29.9	12.2	8.0	5.8
Total P (g)	0.14	0.20	0.12	1.0	1.6	0.50	0.37	0.28
Total C (g)	13.4	21.0	12.7	12.2	19.8	14.1	21.8	12.9
Total N (g)	0.8	0.8	0.3	2.0	2.0	2.3	2.3	0.9
C/N	17.3	27.2	41.8	6.0	9.8	6.0	12.9	24.5

Quantities are given in DW and % of DW. BF, digester feed sludge mixed without sawdust; BFS: digester feed sludge mixed with sawdust; BLFS: low-concentration digester feed sludge mixed with sawdust; BD, digested sludge mixed without sawdust; BDS: digested sludge mixed with sawdust; BN, ammonia removal sludge mixed without sawdust; BNS, ammonia removal sludge mixed with sawdust; BLNS, low-concentration ammonia removal sludge mixed with sawdust

### Analytic methods

#### Chemical analysis

Triplicate samples (5 g each) were dried at 105 °C for 16 h to determine DW, followed by heating at 550 °C for 4 h to calculate FS and VS. CNS-200 elemental analyser was used to determine total carbon (total C) and total nitrogen (total N) based on the manufacturer’s instructions (LECO Corporation, St. Joseph, MI, USA). Total phosphorus was determined in the Environmental Laboratory of the University of Helsinki (Lahti, Finland) as a purchased service according to the standard SFS 3026-230915.

#### Volatile fatty acid analysis

To analyse VFAs, a 200 µL aliquot of the bioreactor supernatant sample was diluted 10-fold with ultrapure water, and 100 µL of 99% formic acid was added to acidify the solution to a pH below 3. The mixture was centrifuged at 20,000 x g for 5 min, and 1.0 mL of the supernatant was transferred to a glass GC vial. The standard series for identification and quantitation of VFAs contained acetic, propionic, butyric, valeric, and caproic acids in concentrations of 10, 50, 100, 250, 500, 750, and 1000 mg/L. Standards and samples were analysed on an Agilent 6890 GC-FID (gas chromatograph with a flame ionization detector) with a ZB-FFAP column (Zebron; 30 m, 0.25 mm internal diameter, 0.25 μm film thickness, Phenomenex, Torrance, California, United States ). The temperature of the injector and detector was both 250 °C, and the injection volume was 1.0 µL. The following program was used: 80 °C as an initial temperature for 4 min, followed by a gradual increase to 140 °C at a rate of 5 °C /min, and finally to 220 °C at 25 °C /min, held for 2 min.

#### Cellulose analysis

Approximately 50 mg of dry sample was used for cellulose and lignin analyses, obtained from 0.5 mL of the mixture in each bioreactor after freeze-drying (Christ Alpha 1–4, Osterode am Harz, Germany). To decompose the lignocellulose, 4 mL of 2% sodium hydroxide was added to the dry sample before autoclaving at 120 °C for 1 h. After cooling, the sample was filtered through a pre-weighed Whatman GF/C glass microfiber filter (GE Healthcare, Buckinghamshire, UK) to separate the solid cellulose on the filter from the alkali-soluble lignin. A sodium hydroxide solution (0.4%) was used for washing until the brown colour of lignin disappeared, and then washing was continued with ultrapure water until the filtrate was neutral. Filters were oven-dried at 105 °C for 16 h to obtain DW before heating for 4 h at 450 °C to determine the fixed solid weight. The analysis was based on the methods presented in Gilarranz et al. (1998), Aldaeus et al. (2011), and Bali et al. (2015).

#### Microbial community analysis

Shotgun metagenomic sequencing was performed to analyse the microbial community of bioreactor samples by Novogene Europe (Cambridge, UK). Samples were collected on the 11th day of BF, BFS, BLFS, the 8th day of BD, BDS, and the 5th day for BN, BNS, BLNS, which corresponded to the maximum VFA yield. DNA extraction was performed using the DNeasy^®^ Power Soil^®^ Kit (100) (Qiagen GmbH, Hilden, Germany) based on the instructions. Frozen (-20 °C) samples of 100–150 µg were used for DNA isolation. The quality and quantity of the DNA extract were verified by agarose gel electrophoresis (1.5%) run at 100 V for 1 h in a TAE-buffer at pH 8.4 (40 mM Tris base, 20 mM acetic acid, 1 mM ethylenediaminetetraacetic acid EDTA). The gel image was taken with a UV-illuminator (Universal Hood II Bio-Rad Gel Doc XR System, Hercules, CA, USA) with ethidium bromide dye fluorescence detection specific for DNA. All these steps were conducted in the Environmental Laboratory of the University of Helsinki (Lahti, Finland). DNA extraction samples were stored at -20 °C before shipping to Novogene Europe for shotgun metagenomic sequencing.

Library preparation and Illumina sequencing were performed by Novogene Europe following their standard shotgun metagenomic workflow. Genomic DNA was randomly fragmented, end-repaired, A-tailed, and ligated with Illumina adapters. Libraries were pooled and sequenced according to the required data output. The relative abundance was manually from Krona figure extracted into an Excel spreadsheet (Supplementary data). Due to the unavailability of raw sequencing reads, analyses such as ASV generation, diversity indices, rarefaction curves, or multivariate statistics were not performed.

#### Statistical analysis

The results were calculated as an average ± standard deviation (S.D.) (*n* = 3). Statistical analyses were performed using IBM SPSS Statistics 29 (New York, USA). The repeated measures ANOVA (RMA) followed by pairwise comparisons (PC) was used to determine whether the amount of sodium hydroxide, and VFA concentration differed in bioreactors at different time points. Two-factor (sludge, bioreactor composition) Kruskal-Wallis (KW) test followed by Mann-Whitney’s test (MW) was used to determine whether sludge and bioreactor composition affected VFA yield.

## Results

### Composition of bioreactors and initial substrate characteristics

VS in sawdust, digester feed sludge and ammonia removal sludge occupied the highest portion of DW of these substrates (Table 1). In contrast, activated sludge, digested sludge, and compost exhibited lower VS contents, ranging from 562 to 634 mg/g. In agreement with VS content, total C contents were highest in sawdust, digester feed sludge and ammonia removal sludge (473–487 mg/g), while activated sludge, digested sludge, and compost contained about 30% of DW for total C. Sawdust contained the lowest amount of total N (0.08 mg/g DW), while ammonia removal sludge, activated sludge, digester feed sludge, digested sludge, and compost contained 27–81 mg/g for total N. As a result, the C/N was extremely high in sawdust (approximately 5835), followed by digester feed sludge (18.1). In contrast, ammonia removal sludge, activated sludge, digested sludge and compost exhibited relatively low C/N ratios, ranging from 4.7 to 8.8.

Differences in substrates resulted in variations in the proportion of VS and FS in the bioreactors (Table 2). VS accounted for 59–96% of the total dry solids, while FS ranged from 4% to 40%. The amount of sawdust added to each supplemented bioreactor was 15.9 g. The VS from sludge was 25 g in BF, BFS, BD, BDS, BN, and BNS, and 9.1 g in BLFS and BLNS. High total phosphorus concentrations (1.0–1.6 g) were detected in bioreactors containing digested sludge, which also showed the highest total N content (2.0 g or higher), together with ammonia removal sludge. Total C content ranged between 12.2 and 21.8 g across all bioreactors, while total N ranged from 0.3 to 2.3 g. Sawdust supplementation increased the C/N, particularly in BLFS, BDS, and BLNS mixtures (Table 2).

### Degradation of cellulose

**Fig. 1:**
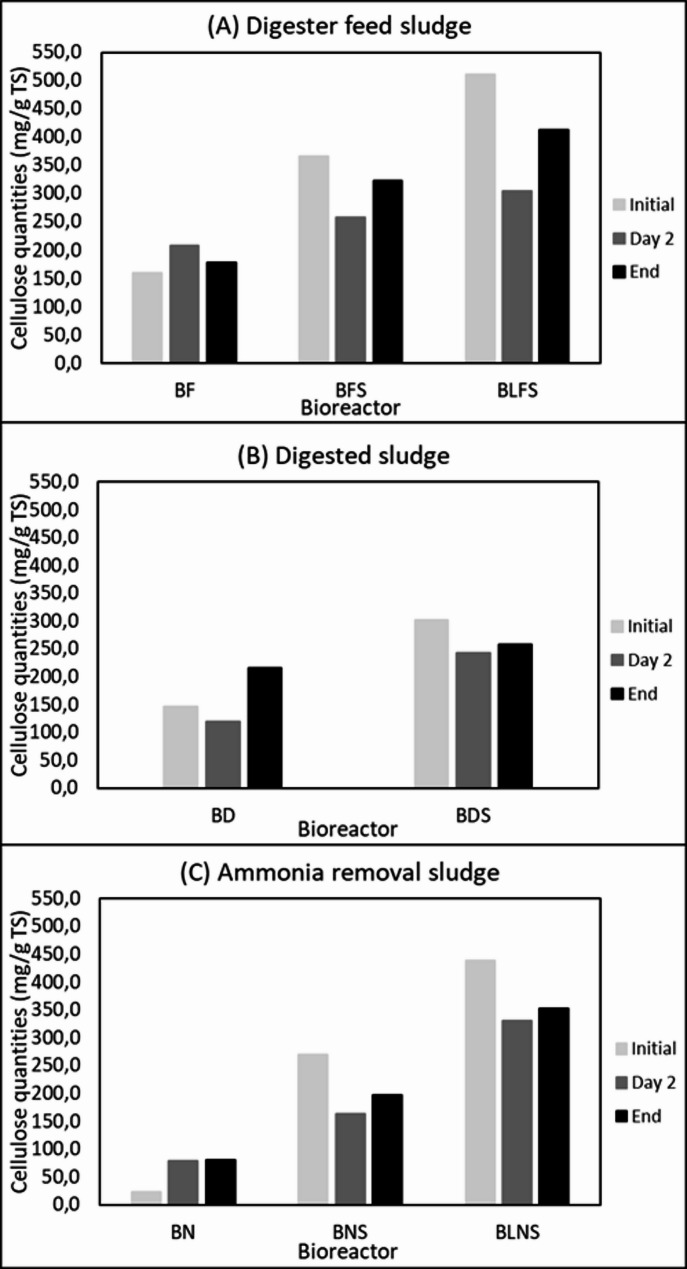
Cellulose quantities (mg/g TS) in bioreactors at the initial stage, day 2, and the end

Cellulose quantities varied depending on bioreactor composition (Fig. 1). Due to the high cellulose content of sawdust (71.9% of DW), bioreactors supplemented with sawdust exhibited higher initial cellulose levels. In bioreactors containing digester feed sludge or ammonia removal sludge supplemented with sawdust, rapid cellulose degradation occurred during the first two days (Fig. 1A and C). After day 2, cellulose concentrations remained relatively stable until the end of incubation period, indicating that cellulose degradation largely ceased after the initial phase (Fig. 1A and C).

### pH adjustment

**Fig. 2:**
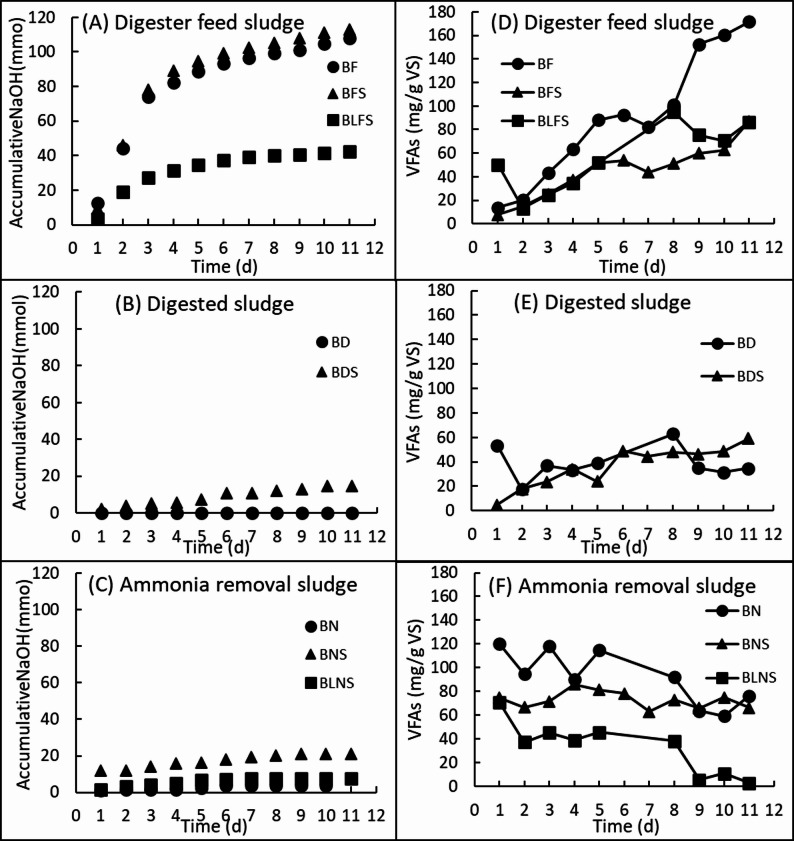
Accumulative consumption of sodium hydroxide (mmole) in bioreactors **A** Digester feed sludge, **B** Digested sludge, **C** Ammonia removal sludge, and the total VFA production (mg/g VS) in bioreactors **D** Digester feed sludge, **E** Digested sludge, **F** Ammonia removal sludge

To maintain a neutral pH (7.0) in all bioreactors, sodium hydroxide (NaOH) was added daily. Bioreactors containing digester feed sludge exhibited a pronounced decrease in pH due to increased acid formation, resulting in substantially higher NaOH consumption, particularly in bioreactors supplemented with sawdust (Fig. 2A). Prior to pH adjustment, pH values in digester feed sludge bioreactors decreased to minimum levels of 4.1 in BF, 4.2 in BFS, and 4.0 in BLFS. In contrast, bioreactors containing digested sludge or ammonia removal sludge required only low amounts of sodium hydroxide maintain pH 7 (Fig. 2B and C). In digested sludge bioreactors, no pH adjustment was required throughout 11-day incubation, as pH values remained stable between 7.1 and 8.0 (Fig. 2B). However, the addition of sawdust to digested sludge (BDS) increased NaOH consumption compared with BD.

### VFA profiles

Daily sampling was performed to quantify VFA production in each bioreactor (Fig. 2D–F). Different bioreactors showed different VFA profiles. In BF bioreactor, VFAs accumulated throughout the 11-day incubation up to 171.6 ± 6.3 mg/g VS (Fig. 2D). Supplementation with sawdust caused a steady and continuing increase in production of VFAs in BFS bioreactor until day 6 and in BLFS bioreactor until day 8 (Fig. 2D). The VFA yields in BFS and BLFS bioreactors were about half of the level obtained in BF bioreactor.

Bioreactors containing ammonia removal sludge showed a different VFA production pattern, characterized by relatively high initial VFA concentrations of 119.9 ± 8.7 mg/g VS in BN and 74.5 ± 5.6 and 70.6 ± 2.5 mg/g VS in BNS and BLNS separately (Fig. 2F). VFA concentrations in all bioreactors containing ammonia removal sludge fluctuated over time, and no clear net accumulation of VFAs was observed during the incubation period (Fig. 2F). In contrast, bioreactors containing digested sludge produced comparatively low amount of VFAs, which was below 62.8 ± 5.2 mg/g VS (Fig. 2E).

**Fig. 3:**
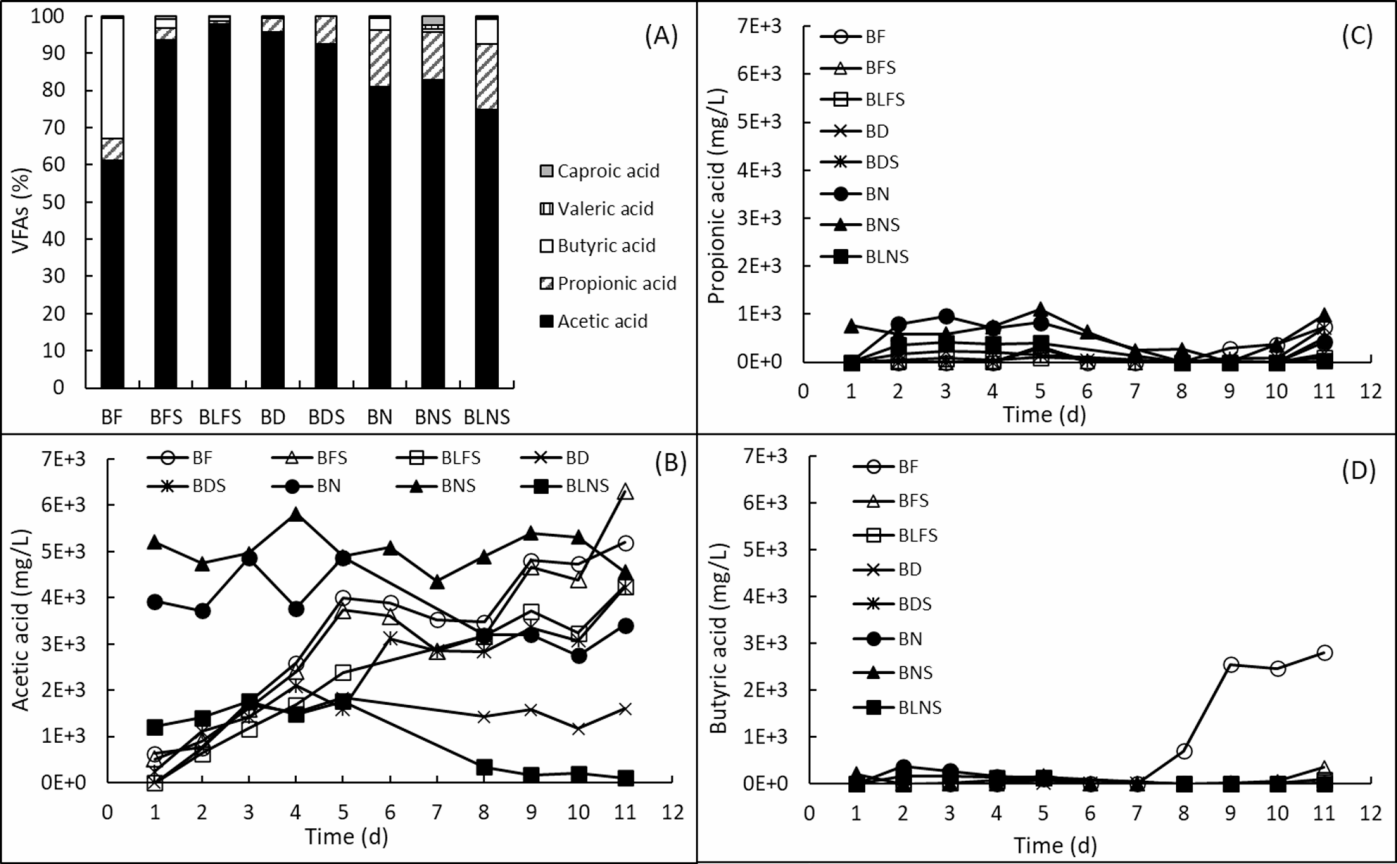
VFA profile in the bioreactors: **A** VFA composition (in percentage of total amount of VFAs) calculated on the average of the three continuing relatively high VFA yields, and accumulation of **B** acetic acid (mg/L), **C** propionic acid (mg/L), and **D** butyric acid (mg/L). Values (mg/L) are given in reference to the substrate volume in each bioreactor. Abbreviations for the substrates: see Table 2

Acetic acid was the most abundant VFA produced in all bioreactors, accounting for 60–90% proportion of the average of the three consecutive high-yield sampling days (Fig. 3A). For BN, BNS, and BLNS, these averages were calculated using data from day 3 to 5, whereas for the remaining bioreactors, data from day 9 to 11 were used. In bioreactors containing ammonia removal sludge (BN and BNS), the concentrations of acetic acid were already high at the beginning of incubation (Fig. 3B). In contrast, in BLNS bioreactor, which contained a lower proportion of ammonia removal sludge combined with sawdust, acetic acid ceased after one week of incubation. In bioreactors containing digester feed sludge (BF, BFS, and BLFS), an increasing trend of acetic acid production was observed, and final concentrations on day 11 reached relatively high levels (Fig. 3B).

Propionic acid was the second most abundant VFA produced in the bioreactors, except in BF and BLFS (Fig. 3C). However, the levels of propionic acid remained below 1 g/L in all bioreactors, with mild increase or decrease profiles in the accumulation of this VFA being observed (Fig. 3C). However, in BF bioreactor, butyric acid was the second most abundant VFA (2609 ± 60 mg/L), accounting for 32.5% of total VFAs (Fig. 3A and D). In other bioreactors, the production of butyric acid was low, and represented a minor fraction of total VFAs. Notably, there was no butyric acid detected in BDS bioreactors (Fig. 3D). Compared to the bioreactors BF, BFS, BLFS, BD, BDS, BN, and BLNS separately, there was no noticeable difference in the valeric and caproic acid concentrations. In these bioreactors, acetic, propionic, and butyric acids comprised in summary over 99%. In BNS bioreactor, the total amount of these three acids was 96.6%. Valeric and caproic acids in all bioreactors occupied less than 0.9% of the produced VFAs, except in BNS, where the percentage of caproic acid in BNS was 2.5%. Overall, sawdust supplementation did not result in a substantial shift from short-chain VFAs toward valeric or caproic acids under the conditions tested.

### Microbial community composition in the bioreactors

**Fig. 4:**
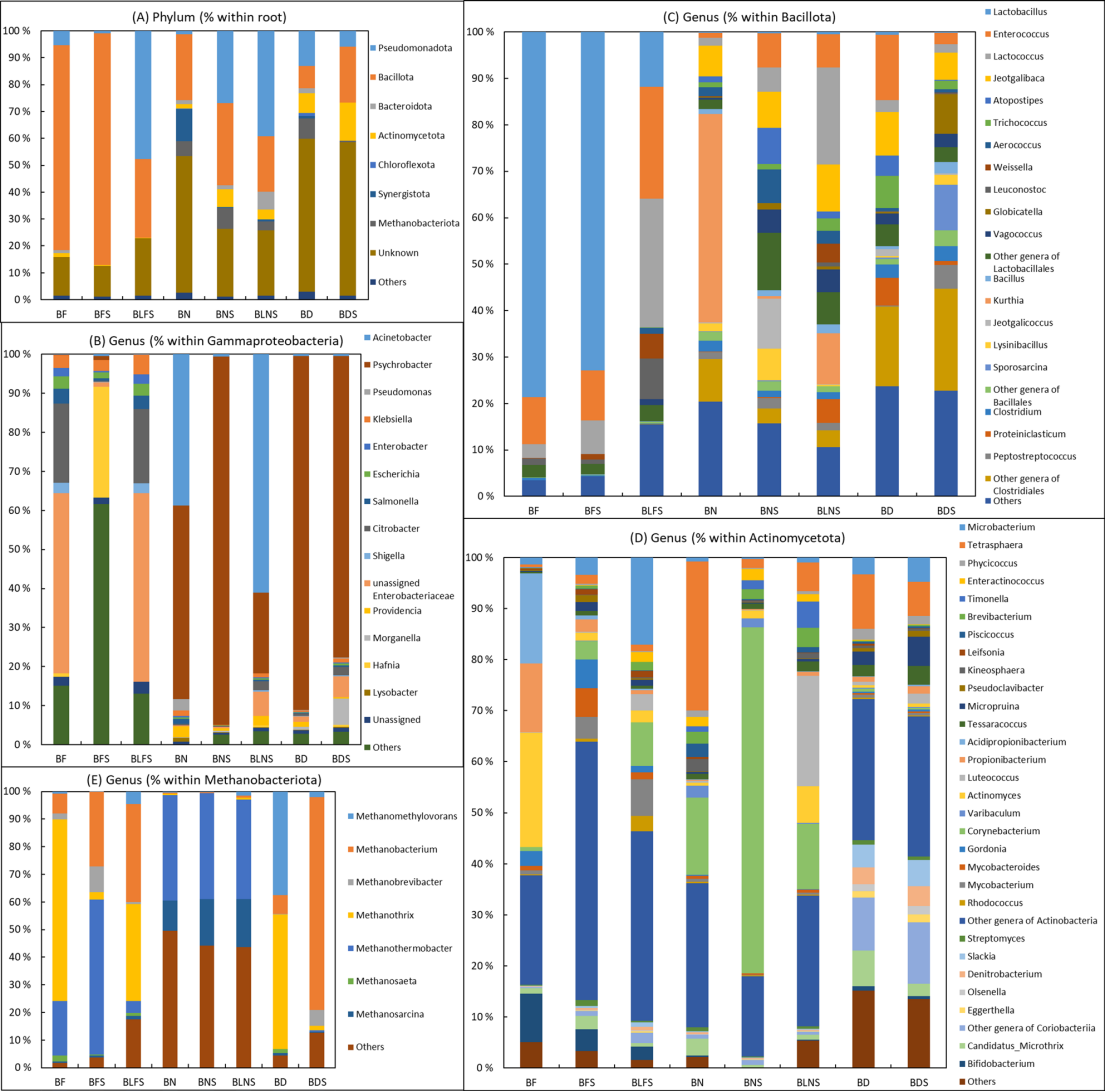
Composition of microbial (prokaryotic) communities in the bioreactors containing different sludges with or without addition of sawdust. Relative abundance at **A** phylum (% within root), **B** genus (% within Gammaproteobacteria), **C** genus (% within Bacillota), **D** genus (% within Actinomycetota), and **E** genus (% within Methanobacteriota) level

The microbial community profile of the bioreactors was characterized using shotgun metagenomic sequencing to assess the effects of different sludge substrates and sawdust supplementation. Relative abundances at the phylum, family and genus levels are presented in Fig. 4. Microbial community composition is presented based on manually extracted taxonomic profiling outputs from Krona figure for bioreactor samples.

Across all bioreactors, seven microbial phyla were detected: Pseudomonadota, Bacillota, Bacteroidota, Actinomycetota, Chloroflexota, Synergistota, and Methanobacteriota (Fig. 4A). Bioreactors containing digester feed sludge (BF and BFS) exhibited similar phylum-level community structures, with Bacillota dominating at 76.2% in BF bioreactor and 86.2% in BFS bioreactor, respectively. In contrast, the relative abundance of Bacillota was markedly lower in BLFS (29.2%), where Pseudomonadota became the dominant phylum (47.7%). A similar dominance of Pseudomonadota (over 50%) was observed in bioreactors containing a higher proportion of sawdust relative to sludge (BLFS and BLNS) (Fig. 4A). This shift was less pronounced in bioreactors containing digested sludge (BD and BDS). In bioreactors containing digested sludge (BD and BDS) and ammonia removal sludge (BN), more than 50% pf operational taxonomic unit (OUT) reads could not be assigned to known phyla. This suggests the presence of less-characterized microbial population, potentially originating from prior anaerobic digestion or fermentation processes associated with these sludge types. Small proportions of Bacteroidota and Methanobacteriota were also detected in these bioreactors, pointing to partially anaerobic microenvironments (Fig. 4A).

At the genus level, distinct community structures were observed (Fig. 4B–E). Within the phylum Pseudomonadota, the class Gammaproteobacteria predominated over Alphaproteobacteria and Betaproteobacteria. In BF and BLFS bioreactors, *Citrobacter* was the most abundant genus within Gammaproteobacteria, accounting for 20.3% and 19.0%, respectively. Other genera showed less than 5% a share of Gammaproteobacteria. In contrast, *Hafnia* dominated Gammaproteobacteria in BFS (28.4%). In BN, *Acinetobacter* (38.9%) and *Psychrobacter* (49.7%) were dominant; however, in BNS, the relative abundance of *Acinetobacter* decreased while *Psychrobacter* became dominant. This shift was not observed in BLNF. In bioreactors containing digested sludge (BD and BDS), *Psychrobacter* accounted for 90.6% and 77.3% of Gammaproteobacteria, respectively.

Within the phylum Bacillota, BF and BFS exhibited similar genus-level profiles, dominated by *Lactobacillus*, *Enterococcus*, and *Lactococcus* (BF: 79.2%, 10.3%, 2.9%; BFS: 73.2%, 10.8%, 7.3%, respectively; Fig. 4C). In BLFS, the relative abundance of *Lactobacillus* decreased, while *Enterococcus* and *Lactococcus* increased to 24.0% and 27.7%, respectively. Bioreactors containing ammonia removal sludge (BN, BNS, and BLNS) displayed a different distribution, with *Kurthia* dominating in BN (44.9%) and the other genera were present between 1.0 and 6.6% within Bacillota (Fig. 4C). Sawdust supplementation in BNS increased the abundance of *Jeotgalicoccus* (to 10.8%) and genera of *Lactobacillaceae*. In BLNS, a more even distribution among *Lactococcus*, *Kurthia*, and *Jeotgalibaca* was observed (20.9%, 10.9% and 10.2%, respectively). Bioreactors containing digested sludge (BD and BDS) showed a relatively even distribution among genera within Bacillota, including notable proportions of *Clostridiales* and *Clostridia.* In BDS, sawdust supplementation increased the relative abundance of *Sporosarcina* and *Globicatella* (Fig. 4C).

Within Actinomycetota (Fig. 4D), BF was dominated by *Actinomyces* (22.4%), *Acidipropionibacterium* (17.7%), and *Propionibacterium* (13.5%). In BFS, a decrease in their relative abundances was noticed while the share of other genera within Actinomycetota increased. In BLFS, *Microbacterium* occupied 17.1% while other genera were below 8.5% in relative abundance among *Actinomycetota*. In BN, *Tetrasphaera* (29.1%) and *Corynebacterium* (15.2%) dominated, while sawdust supplementation in BNS resulted in a marked increase in *Corynebacterium* (68.2%). In BLNS, a more even distribution of genera among Actinomycetota occurred, including *Luteococcus* (21.6%) and *Corynebacterium* (12.7%). The relative abundance of other genera was lower than 7.2%. In BD and BDS bioreactors, *Tetrasphaera* was the most abundant genus (10.6% and 6.7%, respectively), while most were assigned into the less resolved group of other genera of Actinomycetota (Fig. 4D).

Within Methanobacteriota, seven archaeal genera were detected across all bioreactors: *Methanomethylovorans*, *Methanobacterium*, *Methanobrevibacter*, *Methanothrix*, *Methanothermobacter*, *Methanosaeta*, and *Methanosarcina* (Fig. 4E). Their relative abundances varied among bioreactors.

## Discussion

This research systematically compared VFA production from three distinct types of sludges under neutral pH conditions, revealing clear differences in acidogenic performance linked to substrate characteristics. Among the tested waste materials, digester feed sludge showed the greatest potential in terms of produced VFA (mg/g VS). In contrast, no appreciable increase in VFAs was observed in bioreactors fed with digested sludge or ammonia removal sludge. These findings highlight the critical role of sludges in determining VFA production potential. The detailed argumentation in this matter is presented in this section.

### Influence of sludge characteristics on VFA production

The superior VFA yield observed in digester feed sludge bioreactors can be attributed to the high content of biodegradable organic matter, as reflected by high VS and total C values (Li et al. 2018). This sludge originated from a mixture of source-separated biowaste and wastewater sludge and had not undergone extensive stabilization, making it highly susceptible to microbial hydrolysis and acidogenesis. Consequently, a substantial fraction of carbohydrates and proteins remained accessible for microbial hydrolysis and subsequent acidogenesis, facilitating rapid conversion to VFAs (Regueira et al. 2020). The pronounced acidification observed in these bioreactors, together with the high sodium hydroxide demand required to maintain neutral pH, further supports the occurrence of intensive fermentative activity and high VFA formation rates. The maximum VFAs obtained from bioreactors using digester feed sludge was 171.6 ± 6.3 mg/g VS. Similar yield was achieved during the fermentation with heat-alkaline pretreated sludge (240.14 mg COD/g VS, about 171.5 mg/g VS) (Ma et al. 2016).

In contrast, digested sludge is the residual product of anaerobic digestion during biogas production and therefore contains substantially reduced levels of readily fermentable organic matter, with approximately 50% lower VS and total C contents compared to digester feed sludge and ammonia removal sludge (Mottet et al. 2010; Quist-Jensen et al. 2018). The remaining organic matter is largely composed of recalcitrant fractions such as microbial biomass residues and humified compounds, which are less amenable to further hydrolysis. As a result, acidogenic activity was limited, leading to the lowest VFA production potential among the investigated substrates. The relatively stable pH profile in digested sludge bioreactors further indicates low acid formation rates rather than inhibition of fermentation, in agreement with previous findings on post-digestion sludge fermentation. Consequently, digested sludge exhibited the lowest VFA production potential in this study.

Ammonia removal sludge showed relatively high initial VFA concentrations but did not exhibit sustained accumulation. Instead, VFA concentrations decreased or fluctuated over time in BN and BLNS bioreactors. This behavior suggests that VFAs present at the start of incubation likely originated from upstream treatment processes, while the indigenous microbial community favored VFA consumption rather than net production. Sludges derived from nitrification–denitrification systems are typically enriched in aerobic or facultative heterotrophs adapted to organic carbon oxidation and nutrient removal rather than fermentative metabolism, which can promote VFA uptake or conversion to CO₂ rather than net accumulation. In contrast, in BNS bioreactors supplemented with sawdust (16 g), this decreasing trend was not observed, indicating that increased carbon availability may partially counterbalance VFA consumption (Arslan et al. 2017; Ge et al. 2015).

### Influence of sawdust supplementation and hydrolysis limitations of lignocellulosic substrates

Sawdust supplementation increased the VS fraction in all bioreactors, particularly in BD and BDS bioreactors. However, this increase in VS did not lead to enhanced VFA production obviously, indicating that VS content alone is not a reliable predictor of acidogenic performance. Although sawdust is rich in cellulose, hemicellulose, and lignin, its lignocellulosic structure severely limits bioavailability under mild aerobic conditions. Lignin can physically shield cellulose and hemicellulose, preventing direct microbial hydrolysis. Without pretreatment methods such as thermal, chemical, or oxidative processes, sawdust cannot serve as an effective carbon source for rapid VFA production.

These results align with previous studies showing that lignocellulosic residues require physicochemical pretreatment to become effective substrates for acidogenic fermentation (Arslan et al. 2017; Wu et al. 2016). The rapid cellulose degradation observed during the initial incubation phase suggests that only the most accessible carbohydrate fractions were utilized, after which hydrolysis became rate-limiting. From a bioprocess perspective, this highlights the need for pretreatment strategies or co-substrate selection when targeting VFA production from lignocellulosic wastes.

### Role of pH control in VFA production

Maintaining neutral pH was essential for stable VFA accumulation across all bioreactors. pH plays an important role in VFA production because it influences the balance between acidogenic and methanogenic microorganisms (Varghese et al. 2022). Although it has been considered that the costs associated with the use of chemicals were needed for pH control, a neutral pH has proven to be the most favorable for producing VFAs from organic matter (Castro-Fernandez et al. 2024). Digester feed sludge bioreactors required higher NaOH dosages due to rapid acid formation, reflecting strong fermentative activity and continuous VFA accumulation. In contrast, digested sludge and ammonia removal sludge required minimal adjustment of pH and produced lower VFA yields, suggesting reduced acidogenic potential. These findings confirm that pH regulation is a critical operational parameter in aerobic sludge-to-VFA bioprocesses and should be optimized alongside substrate selection to balance process efficiency and chemical consumption.

### VFA composition and process implications

Acetate is often the major component of the total VFA, which was also observed in the present study. Acetate comprised 61–98% of the total VFAs. In bioreactor BF, butyrate was the second largest VFA component, while propionate was the second dominant in bioreactors using digested sludge and ammonia removal sludge. In BFS and BLFS, the portion of butyrate and propionate was close. The present results are consistent with previous studies, which observed VFA production by a granular sludge process. Acetate and butyrate were the main products (Tamis et al. 2015) as the results in BF. The result was consistent with the results from previous studies, which found that acetic acid was dominant at neutral pH (Cokgor et al. 2009) and at room temperature (Ahn and Speece 2006).

The predominance of even-carbon VFAs, particularly acetic and butyric acids, is advantageous for downstream bioprocesses such as PHA production, where these acids serve as preferred precursors for 3-hydroxybutyrate synthesis (Hao et al. 2017). However, the limited formation of valeric and caproic acids indicates that chain-elongation pathways were not favoured under the applied conditions, suggesting opportunities for process optimization if odd-carbon VFAs are desired.

### Microbial community structure and functional relevance

Pseudomonadota and Bacillota are famous fermentative bacteria and exist widely in the anaerobic digestion process (Díaz et al. 2018; Lee et al. 2012; Tyagi et al. 2021) and are VFA producers (Atasoy et al. 2019; Jankowska et al. 2018). These two phyla occupied 76.9–87.1% of the microbial community in bioreactors using digester feed sludge, which played a key role in VFA production and supported the relatively high VFA yield in these bioreactors. Among Pseudomonadota, Gammaproteobacteria comprised the most significant part, occupying 89.5–98.9% of Pseudomonadota. Genera related to VFA production within Gammaproteobacteria includes *Citrobacter*, members of which have also been applied as VFA-producing organisms (Yang et al. 2015). Genus *Citrobacter* was apparently the main VFA producer within Pseudomonadota in BF bioreactor. The genera have the function on cellulose degradation, including *Acinetobacter* (Li et al. 2022), *Pseudomonas* (Sun et al. 2020), *Klebsiella* (Korsa et al. 2022), *Enterobacter* (Sari et al. 2017), *Escherichia* (Pang et al. 2017), *Citrobacter* (Flimban et al. 2019), *Shigella* (Wang et al. 2011) and *Morganella* (Alberoni et al. 2023). *Psychrobacter* plays a key role in the initial degradation of cellulose (Li et al. 2013), which supports the decrease in cellulose in BNS, BLNS, BD, and BDS bioreactors.

Out of these genera belonging to Bacillota, members of *Lactobacillus*, *Enterococcus*, *Lactococcus*, and *Clostridium*, have also been reported to produce VFAs (Fang et al. 2022; Jin et al. 1998; Ma et al. 2019; Pang et al. 2014). *Lactobacillus*, as the most abundant genus in BF and BFS bioreactors, is a gram-positive, anaerobic bacterium (Lau et al. 2004), which was also clearly dominant where VFA concentrations were higher (Rasi et al. 2022). This genus may be responsible for significantly increasing the concentration of total VFAs (Jin et al. 1998), resulting in the maximum VFA yield obtained in BF. The addition of *Enterococcus*, as the second most abundant genus in BF, BFS, and BLFS, increased total VFA production (Pang et al. 2014). *Lactococcus* could produce high levels of acetic, propionic, and butyric acids (Fang et al. 2022). Members of the genus *Bacillus* have been widely studied for PHA production using VFAs, including acetic acid (Munir and Jamil 2018), propionic acid (Munir and Jamil 2018), and butyric acid (Shahid et al. 2013). Out of these genera, members of *Enterococcus* (Wang et al. 2009), *Atopostipes* (Vogel et al. 2024), *Bacillus* (Deng and Wang 2022; Wang et al. 2024), *Lysinibacillus* (Lu et al. 2025), and *Clostridium* (Van Dyke and McCarthy 2002) are related to cellulose degradation, which supported the initial cellulose degradation in bioreactors BFS, BLFS, BNS, BLNS, BD, BDS.

Within the phylum Actinomycetota, *Acidipropionibacterium* and *Propionibacterium* belong to the propionic acid bacteria, which can produce propionic acid (Bücher et al. 2021). *Bifidobacterium* has the ability to produce acetic acid (Dicks and Botes 2010). Additionally, *Bifidobacterium* is involved in butyrate production (Fernández-Navarro et al. 2018), because it was the product of the secondary fermentation of the acetate (Duncan et al. 2004; Rios-Covian et al. 2015). *Olsenella* could produce acetic acid from fermented starch and glycogen substrates (McLoughlin et al. 2020). The genera related to cellulose degradation within the phylum Actinomycetota include *Brevibacterium* (Yang et al. 2024) and *Rhodococcus* (Ma et al. 2024; Yasin et al. 2023).

### Implications for sludge valorisation and bioprocess development

From a bioprocessing perspective, the results demonstrate that sludge with high biodegradable organic content is a suitable feedstock for VFA production at ambient temperature. Although untreated sawdust did not substantially enhance VFA yields, the study provides important insights into substrate limitations and microbial interactions relevant for process design.

The laboratory-scale tests of VFA-production bioreactors provide a foundation for pilot-scale as well as industrial-scale implementation and integration into wastewater treatment plants as a resource recovery strategy. Sludge with high organics is a good material for VFA production, although even-carbon VFAs, such as acetic and butyric acids, were the main products. These were the source for the biosynthesis of 3-hydroxybutyric acid (Hao et al. 2017). There are additional processes that await exploration and could further enhance the production of odd-carbon VFAs, such as propionic and valeric acids. Future studies should focus on pretreatment of lignocellulosic co-substrates, microbial community steering, and coupling VFA production with downstream bioproduct synthesis, such as PHA accumulation, to enhance the overall sustainability and economic feasibility of sludge-based bioprocesses.

## Conclusions

This study systematically evaluated the potential of three different wastewater-derived sludges for VFA production under controlled laboratory-scale conditions. Among the tested substrates, digester feed sludge demonstrated the highest VFA yield (171.6 ± 6.3 mg/g VS), highlighting its suitability as a feedstock for VFA-oriented bioprocesses. In contrast, digested sludge and ammonia removal sludge exhibited limited VFA accumulation, likely due to their lower biodegradable organic content and higher degree of prior stabilization. The addition of sawdust increased the VS content but did not significantly enhance VFA production, indicating that untreated lignocellulosic material was not readily bioavailable under the applied operating conditions. Across all bioreactors, acetic acid was the dominant VFA, accounting for 61–98% of total VFAs, which is favourable for downstream bioprocessing applications such as hydroxybutyrate production. Propionic and butyric acids were the other dominant acids. More valuable acids, such as valeric and caproic acid, were present in extremely low percentages, which could be explored further to improve their concentrations. Microbial community analysis revealed the dominance of Bacillota and Pseudomonadota, with *Lactobacillus* being particularly abundant in bioreactors exhibiting high VFA yields, suggesting a functional link between microbial composition and fermentation performance. Overall, the findings demonstrate that sludge type is a critical determinant of VFA production efficiency and that substrates rich in readily biodegradable organics offer the greatest potential. It remains unknown whether scaling up the VFA production system to a pilot-scale reactor affects VFA production and composition. The study can serve as a reference for full-scale applications of VFA production in WWTPs, aiming to reduce operational costs. Besides, these results provide a scientific basis for the selection of suitable sludge streams for VFA recovery and support further investigation at pilot scale, including process optimization and integration with downstream bioprocesses.

## Data Availability

All data are included within the manuscript and its Supplementary Material. The Excel file containing relative abundance data is provided as Supplementary Material. Raw sequencing files are unavailable. Additional supporting information is available from the corresponding author upon reasonable request.

## Abbreviations

BD: Digested sludge mixed without sawdust
BDS: Digested sludge mixed with sawdust
BF: Digester feed sludge mixed without sawdust
BFS: Digester feed sludge mixed with sawdust
BLFS: Low-concentration digester feed sludge mixed with sawdust
BLNS: Low-concentration ammonia removal sludge mixed with sawdust
BN: Ammonia removal sludge mixed without sawdust
BNS: Ammonia removal sludge mixed with sawdust
C/N: Carbon-to-nitrogen ratio
DW: Dry weight
PHA: Polyhydroxyalkanoate
VFA: Volatile fatty acids
VS: Volatile solids
WWTPs: Wastewater treatment plants
